# 2764. Experience with dalbavancin in a pediatric hospital

**DOI:** 10.1093/ofid/ofad500.2375

**Published:** 2023-11-27

**Authors:** Maria Deza Leon, Mark Murphy

**Affiliations:** Cincinnati Childrens Hospital, Cincinnati, Ohio; Cincinnati Childrens Hospital, Cincinnati, Ohio

## Abstract

**Background:**

Due to its long half-life allowing for once-weekly dosing, dalbavancin has become an appealing option for the treatment of infections that require prolonged antibiotic courses. While studies have shown non-inferiority of dalbavancin compared to conventional antibiotic regimens, there is limited information regarding its use in the pediatric population. We describe the experience with dalbavancin use in a large pediatric hospital.

**Methods:**

Children and young adults who were seen at Cincinnati Children’s Hospital between the period of July 1st, 2019 and December 31^st^, 2022 and received antibiotic therapy with Dalbavancin were included in this study. We reviewed the patient’s demographic data, laboratory findings including microbiologic data, clinical course, response to treatment and outcomes. Treatment failure was defined as no response or worsening after initial improvement. Clinical response was also evaluated at day 90 after end of therapy.

**Results:**

We identified 26 children and young adults who received dalbavancin between July 1^st^ 2019 and December 31^st^ 2022 (Table 1). Ages ranged between 6 months and 22 years. Most common indication was osteomyelitis. Side effects were rare and included flushing and transient vision loss, which was later diagnosed as ocular migraine. Seven patients (26.9%) had treatment failure at 90 days, including 33.3% and 18.2% of patients with and without comorbidities, respectively. Ages of patients with treatment failure ranged from 6 months to 20 years. Five of them showed initial improvement during the first week after therapy but required an antibiotic switch due to worsening. Time to failure from first dose of dalbavancin ranged from 10 to 35 days. Of all 7 patients who failed therapy, four had no recovered organisms, two had *MRSA* and one had *MSSA*. One patient with no initial recovered organism grew Pseudomonas aeruginosa on a repeat culture.

Characteristics of patients who received dalbavancin between July 1st, 2019 and December 31st,2022

**Figure:**
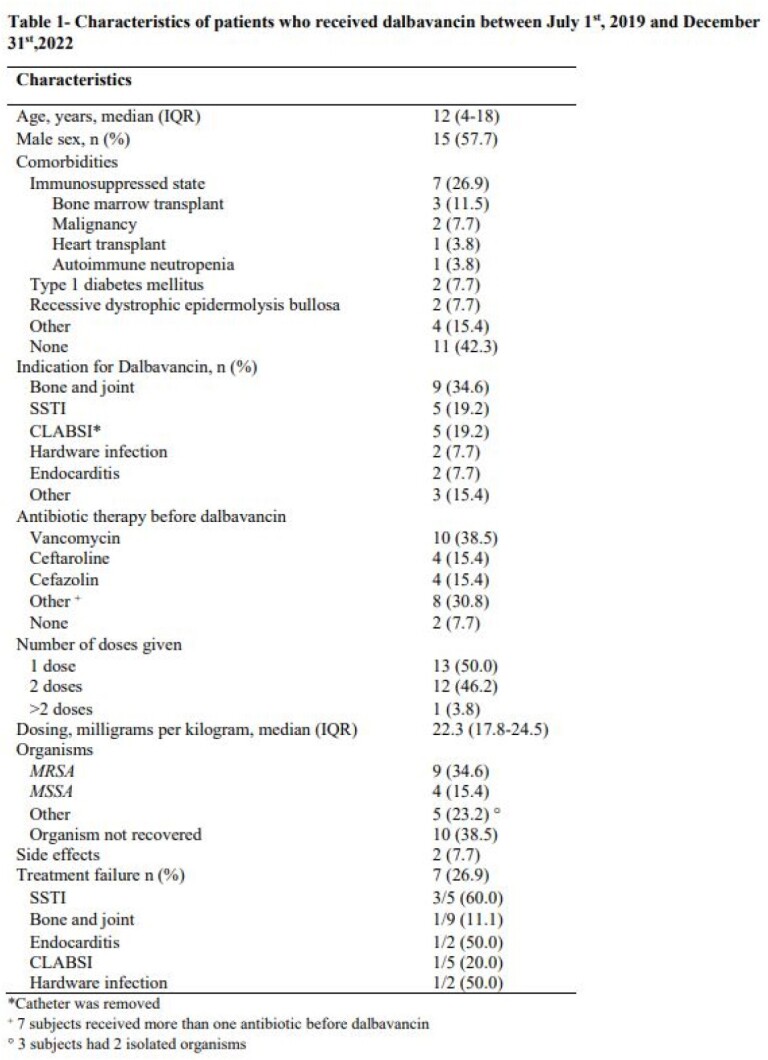

**Conclusion:**

While our population was small, a high treatment failure rate was seen when using dalbavancin, particularly in patients with comorbidities. More data is needed to evaluate its performance in the pediatric population and clinicians should be thoughtful about deciding who is a good candidate for dalbavancin therapy.

**Disclosures:**

**All Authors**: No reported disclosures

